# Materia Medica and Therapeutics

**Published:** 1848-01

**Authors:** 


### BIBLIOGRAPHICAL NOTICES.

*Materia Medica and Therapeutics.* By MARTYN PAINE, A. M., M. D., Professor of Institutes of Medicine and Materia Medica in the University of New York, &c., &c. 12mo. pp. 412. New York: 1848.

The Author of this work is one of the most laborious, but not most perspicuous or successful writers of the country. Yet, although his productions can scarcely have received that pecuniary recompense, which, notwithstanding the mawkish sentimentality of some, is a sweet solace to exertion, he perseveres in endeavouring to render himself useful; and his present production is certainly calculated to be much more so than the "Therapeutical Arrangement of the Materia Medica," on which we found it necessary to animadvert a few years ago. Both, however, are too much skeletons or syllabuses to be extensively used out of the Author's class; and except in that locality, the constant reference made to his other works, and especially to his latest—"the Institutes of Medicine"—must still more restrict their adaptation.

Dr. Paine has long hoisted his flag—nailed it, indeed, to the mast—as a vitalist: and is hence but little disposed to credit the action of chemical agents on the living economy. The following motto at the very commencement of the work is, indeed, ominous on this point. "It is only," says Dr. Paris, "here necessary to caution the practitioner against those fallacies into which the captivating theories of the chemist may seduce him." p. 10. Dr. Paine's fourth "general principle is laid down as follows:—

"Remedial agents operate directly upon the vital properties of the parts to which they are applied, and, through the medium of those parts, upon remote organs, by the principle of sympathy. The partial absorption of certain remedies is only a contingent result, and has little or no agency in the physiological phenomena. Their reputed absorption is greatly overrated, often only imaginary, and sometimes misrepresented. Such as have no natural relation to the vital properties modify the natural condition of the absorbing vessels before they can enter the circulation." p. 10.

We are glad that the author admits that remedies may enter the circulation, and that he does not belong to the *few* who are of opinion, that no extraneous matter can be received into the blood—not even the smallest quantity of milk or mucilage—without exciting disastrous results;—who think, that a true digestion or decomposition must be effected upon them before they can form part of the circulating current; and all this, notwithstanding that substances have for ages been thrown into the blood-vessels without such results, and that others, which have passed into the vessels by endosmotic or other action, have actually been detected there by appropriate reagents. Nor will it do for the author of the work before us to express his disbelief of the extensive absorption of numerous agents administered therapeutically and as poisons. We were amongst those, who once thought, that certain poisons act so instantaneously, that there is not time for them to reach the vital organs, so soon is life destroyed; and we were, therefore, compelled, under the statement of facts, to believe, that the first poisonous impression must have been made on the nerves of the part with which the poison was made to come in contact, whence it extended to other parts of the economy. More accurate experiments have, however, changed our views on this matter; and demonstrated to us, that in every case apparently there is an interval sufficient to permit the poison to pass to the organ on which its deadly influence has to be exerted, before such influence is perceptible. The experiments of Hering, Poiseuille, Matteucci, Blake and others, have sufficiently exhibited the almost inconceivable velocity of the circulation, and thus removed many difficulties from the path. Hering injected a solution of prussiate of potassa into the jugular vein of a horse, and at the same moment drew blood from the opposite jugular. Matteucci did the same; and changed the vessel that received the blood every five seconds. He found, as Poiseuille and Hering had done before him, that the blood, which issued twenty or twenty-five seconds after the injection, presented traces of the prussiate

Similar experiments, with like results, were made by Dr. Blake, formerly of London, now Professor of Surgery in St. Louis. These appeared in the 149th number of the *Edinburgh Medical and Surgical Journal;* and have been again published by Dr. Blake in the *St. Louis Medical and Surgical Journal* for November last.

The substance with which the greatest number of experiments has been made, and which has been supposed to destroy life instantaneously, when applied even in small quantities to a mucous surface, is the concentrated hydrocyanic acid. It has been asserted over and over again in the books, that a drop or two, applied to the conjunctiva of a dog, will instantaneously kill. To test this assertion, Dr. Blake "prepared half a drachm of the anhydrous acid, by passing dried sulphuretted hydrogen over bicyanide of mercury." The whole of this acid was poured on the tongue of a large dog, the head being kept elevated and the mouth held open. Not until eleven seconds did symptoms of the action of the poison appear. The respiration then became affected, and the animal was dead in thirty seconds from the time the poison was applied to the tongue.

Dr. Blake obtained from Sir Benjamin Brodie a portion of the deadly *woorara* poison, and injected five grains of it, dissolved in water, into the jugular vein of a dog: no symptoms of the action of the poison showed themselves until twenty seconds after it had been injected. Violent tetanic convulsions then came on, and the animal died about forty-five seconds after the injection. Dr. Blake infers from all his experiments, that they afford "conclusive evidence, that no objection can be raised against the theory of the absorption of poisons, on the ground, that the circulation is not sufficiently rapid for the blood to carry them to the organs affected in the time that has been observed to elapse between their application and the appearance of the first symptoms of their action." We confess we are every day more and more disposed to believe in the operation of medicines by absorption; and most assuredly we shall never invoke the nervous doctrine until we find that our facts in regard to the rapidity of the circulation are "false," which is not likely to happen. The old method of estimating the velocity of the circulation by taking the whole quantity of blood in the body, the amount sent out at each contraction of the ventricle, and the number of pulsations in a given time, furnished an estimate, which, although somewhat surprising, appears to be far below the fact. If we value the quantity of blood in an average sized person at thirty pounds, the amount sent out during the systole of the ventricle at two ounces, and the number of pulsations in the minute at 80, the whole of the fluid of the circulation must pass through the heart in three minutes; and if we assume the estimate of the capacity of the ventricle made by Valentin to be correct—five ounces—it would be distributed in one and one-fifth of a minute. We do not then accord with the author, that the "reputed absorption of remedies is greatly overrated, often only imaginary, and sometimes misrepresented."

On a former occasion we spoke of the author's classification of therapeutical agents. We are not more favourably impressed with it now. Who can conceive of a class of "genito-urinary agents!"

We have not space to examine many points which might admit of discussion, and on which we by no means agree with the author. We wish much, that he had followed the nomenclature of the Pharmacopœia of the United States. The authority is good—equal, certainly, to that of any of the foreign pharmacopœias—and intended to be a standard *here* at least. Yet it is rarely followed by him.

Several typographical errors have struck us; and some orthographical inaccuracies, such as hyosciamus, which occurs repeatedly. Speaking of the work *positively*, we can recommend it as a brief epitome of Materia Medica and Therapeutics; and *relatively*, we deem it a great improvement on his former work of a similar kind.

